# COVID-induced 3  weeks’ treatment delay may exacerbate breast cancer patient’s psychological symptoms

**DOI:** 10.3389/fpsyg.2022.1003016

**Published:** 2022-11-10

**Authors:** Yijia Wang, Yuqing Yang, Changjiao Yan, Wen Ma, Jixin Yang, Hongliang Wei, Nanlin Li

**Affiliations:** ^1^Department of Psychology, Colorado College, Colorado Springs, CO, United States; ^2^Department of Thyroid, Breast and Vascular Surgery, Xijing Hospital, Air Force Medical University, Xi’an, Shaanxi, China

**Keywords:** COVID-19, breast cancer, treatment delay, mental health, cancer care

## Abstract

The delayed access to cancer treatment due to the outbreak of COVID-19 pandemic posed a unique challenge to breast cancer patients and caused a significant level of mental distress among them. In the current research, we examined the psychological impacts of COVID on a subpopulation of breast cancer patients from a hospital in Shaanxi province of China using Symptom Checklist-90-R (SCL-90-R). Participants were 195 breast cancer patients at the outpatient clinic of Xijing hospital, Xi’an, Shaanxi Province, China. We found that a treatment delay of more than 3 weeks may exacerbate breast cancer patients’ psychological symptoms, such as somatization, obsessive–compulsive disorder, interpersonal sensitivity, depression, hostility, phobic anxiety, paranoid ideation, and psychoticism, whereas a short-term delay of less than 3 weeks is less likely to have a significant effect on one’s mental well-being. Additionally, breast cancer survivors, especially those at more advance stages, tend to experience more elevated psychological symptoms with longer treatment delay, and whose treatments continues to be delayed reported stronger psychological symptoms than individuals whose treatment are resumed, regardless of treatment type.

## Introduction

Coronavirus-19 (COVID), first reported in Wuhan, China, began as a viral infection in late 2019 and developed into a global pandemic in March 2020. In response to the rampant spread of the virus, the central government of China imposed a strict nationwide lockdown, including the suspension of public transportation and temporary closure of schools, government offices, and factories (Wei et al., 2021). In addition, due to the strong transmission capacity and the high mortality rate of 12% in the epicenter at the early stage of its outbreak (Mizumoto and Chowell, 2020), COVID put unprecedented strain on the Chinese healthcare system.

At the peak of the pandemic, as a direct consequence of the surge of COVID cases, the Chinese government imposed strict lockdowns in various regions, which led to the shortage of healthcare workers, scarcity of healthcare resources, delays in non-emergency medical access, and temporary disruption of cancer care (Czeisler et al., 2020; Sun et al., 2021). Numerous studies have reported delays in all aspects of cancer care and treatments, including screening, diagnosis, surgery, and follow-up visits (Li et al., 2020; Papautsky and Hamlish, 2020). The mental distress caused by the COVID lockdown, coupled with the existential concerns raised by delayed access to life-sustaining treatment, increase cancer patients’ risks of experiencing adverse mental health impact.

COVID posed a unique challenge to breast cancer patients. Empirical research suggests that breast cancer patients, compared to healthy controls, are more likely to experience psychosocial distress and self-esteem crisis due to body image disruption (Pan et al., 2014), and they may develop a stronger need for positive affirmations to alleviate body image anxiety. However, social distancing measures and quarantine policies hamper close interpersonal contacts and potentially reduce one’s likelihood of obtaining positive affirmations, which may in turn cause mental distress. Also, research has shown that cancer survivors may experience feelings of uncertainty even after years of diagnosis due to fear of recurrence (Karlsson, 2014), which increases their chance of depression and anxiety (Parker et al., 2013). The pandemic has brought a substantial level of uncertainty to people worldwide. This uncertainty might be particularly intense for breast cancer survivors whose pre-COVID life following cancer diagnosis had already been inundated with unpredictability.

Approximately 30% of breast cancer patients experienced mental distress before the advent of COVID (Fradelos et al., 2017; Hajian et al., 2017). Given that COVID tends to evoke existential anxiety and terror among the general public (Pyszczynski et al., 2021), some breast cancer patients may be exposed to more substantial psychological stress than pre-COVID level because the virus reminds them of vulnerability and mortality. In addition, research suggested that COVID indeed put cancer patients’ lives at stake. For example, a recent study has found that from January 2020 to February 2020, cancer patients in Hubei, Wuhan, the epicenter of the COVID pandemic, had nearly a threefold higher fatality rate from COVID than healthy individuals (Dai et al., 2020), and the efficacy rate of vaccination for cancer survivors who are immunocompromised might still be lower than non-cancer individuals (National Cancer Institute, 2021). The fact that breast cancer patients are likely to have an increased risk of severe illness and fatality may further exacerbate their existential terror. Thus, void of interpersonal contact and uncertainty, in addition to existential concerns, feeling of vulnerability, and the high mortality rate of COVID, are potentially risk factors for psychopathology during COVID.

Symptom Checklist-90-R (SCL-90-R) has by far been one of the most widely used psychometrics to measure individuals’ general psychopathology (Tian et al., 2020), and extensive studies have found satisfactory psychometric reliability and validity of the scale (Prinz et al., 2013; Carrozzino et al., 2016; Chen et al., 2020). Empirical research demonstrated a positive association between breast cancer status and general psychopathology based upon the SCL90-R factor scores (Pan et al., 2014). Studies also used SCL-90-R factor scores to measure the effect of COVID on the mental wellbeing of the general public. For example, Chinese nationals reported higher SCL-90-R factor scores from January 2020 to February 2020, when all cities were under partial lockdown and public facilities were closed, than the pre-COVID culture norm (Tian et al., 2020).

However, despite that some previous research examined the effects of COVID on the general psychological status of Chinese breast cancer patients (Bartmann et al., 2021; see also Cui et al., 2020; Li et al., 2020), to the best of our knowledge, no research thus far has investigated how molecular types, cancer stages, treatment status, and the length and types of treatment delay individually and collectively impact the psychological health of breast cancer patients. Severe mental distress as result of the interplay between COVID lockdown, existential anxiety, and delay of life-saving cancer treatment may become serious threats to breast cancer patients’ physical and mental wellbeing. Therefore, it is imperative for psychologists and clinical researchers to identify the psychological impacts of treatment delay on breast cancer patients at various cancer stages with different molecular types and develop coping strategies to help them maintain psychological wellbeing during trying times such as COVID. In an effort to fill in the research gap, this study aims to a) evaluate the psychological wellbeing of a subpopulation of breast cancer patients from Xi’an, Shaanxi Province, following the surge of COVID outbreak when their city was under partial lockdown, healthcare facilities were closed, and cancer care was temporarily disrupted, and b) examine how treatment status, cancer stage, length and type of treatment delay, and patients’ specific molecular type independently and interactively affect breast cancer patients’ mental health.

## Materials and methods

### Subjects and study design

The study utilized a cross-sectional design, and the participants were breast cancer patients of different cancer stages at the outpatient clinic of Xijing hospital, Xi’an, Shaanxi Province, China. Due to the social distancing requirements during COVID, the study was conducted virtually, and the questionnaires were distributed to the participants *via* Wenjuanxing, the Chinese alternative of Qualtrics. The researchers were healthcare workers affiliated with Xijing hospital. The survey link was sent to a breast cancer patient WeChat support group which included 1,399 cancer patients and 6 healthcare workers. The patients were told that the study aimed to examine the effect of COVID on their mental health and the status of their cancer treatment, and they could voluntarily choose whether they would like to participate in the study or not. The initial sample consisted of 199 breast cancer patients who previously obtained treatment at Xijing hospital but whose treatments were delayed due to COVID lockdowns. All participants signed an informed consent form that described the purpose of the study and the fact that if they agreed to participate in the study, their responses would be analyzed for clinical research, and their identifying information would be collected but kept confidential. The inclusion criteria were as follows: (a) diagnosed with breast cancer, (b) aged 18 years or above, (c) able to read and comprehend simplified Chinese. The exclusion criteria were as follows: (a) patients with severe cognitive impairment (i.e., retrograde and anterograde amnesia), (b) previous diagnosis of psychiatric disorders (i.e., depression, schizophrenia, or post-traumatic stress disorder), or (c) multiple organ dysfunction syndrome. After eliminating participants who provided partial response or withdrew from the study, the final sample was comprised of 195 individuals.

The study was approved by the Institutional Review Board (IRB) of Xijing hospital, and the goal of the IRB is to prevent harm and protect patients’ rights.

### Survey tools and survey method

Upon signing the informed consent form, participants answered questions regarding their age, marital status, education level, general health status, breast cancer stage, molecular type, and the duration of treatment. Then, participants reported whether there was a direct effect of COVID on their city on a two-point dichotomous scale, followed by a set of questions regarding the length and type of treatment delayed due to COVID as well as the current status of treatment. The treatment types included surgery, adjuvant/neoadjuvant therapy ± target therapy, endocrine therapy, target therapy only, radiotherapy, advanced therapy, and routine check-ups. The current status of treatment was measured on a two-point scale: continue to be delayed and resumed. The molecular types are classified as HR+ HER2−, HER2+, and triple negative.

The validated Mandarin version of the SCL-90-R (Wang, 1984) was then given to the participants to evaluate their psychological status. The SCL-90 checklist is a self-report psychometric instrument that studies one’s subjective symptoms of psychopathology, consisting of 90 items from nine symptom dimensions: somatization, interpersonal anxiety, obsessive–compulsive, depression, anxiety, hostility, phobic anxiety, paranoid ideation, and psychoticism (Sereda and Dembitskyi, 2016). Seven additional items measured participants’ sleep and appetite disturbance. Each dimension was measured on a five-point Likert-type format (1 = not at all, 5 = extremely). Previous studies consistently reported that SCL-90-R is a valid inventory, with adequate construct validity and high factorial validity (see Ransom et al., 2010; Ardakani et al., 2016; Sereda and Dembitskyi, 2016). Additionally, according to our data, the scale was highly reliable and internally consistent (*α* = 0.92). Thus, we computed the mean of all subscales into the overall index of psychological symptoms, known as the general psychopathology score.

### Statistical analysis

Categorical variables were summarized as numbers and percentages; continuous variables were described as mean (*M*) ± standard deviation (*SD*). Data were analyzed using IBM SPSS Statistics Version 26. Independent samples t-test and one way ANOVA were used to study the effect of breast cancer stage, molecular type, current status of treatment (delayed v resumed), and length and type of treatment delayed on general psychopathology score. ANOVA tests were then conducted to examine the main effect and interaction effect of the breast cancer stage, length of treatment delayed, molecular types, and the current status of treatment on SCL-90-R general psychopathology score. Additionally, multiple linear regression analyses were applied to explore the predictive effect of length of treatment delay and breast cancer stage on SCL-90-R dimensions. Each model was adjusted for age, education, perceived health status, breast cancer stage, molecular type, Covid effect on one’s city, current status of treatment, and length of delay in treatment. The *p* values were two-tailed and the statistical significance level was at *p* < 0.05.

## Results

### Demographic characteristics and health status

Participants (*N* = 195) filled out the SCL-90 questionnaire in February, 2020. All participants were female breast cancer patients treated at Xijing hospital, Xian, China, among which 16.41, 36.41, 19.49, and 28.21% had, respectively, received treatment for less than a year, 1–3 years, 3–5 years, and 5 years or more. 64.62% of the patients were at Stage I (the cancer cells stay at their original place); 30.77% were at Stage II and III (the cancer cells are spread to armpits and clavicle but has not reached the bone). 4.62% were at Stage IV (the cancer cells are spread far away to the bones, livers, lungs, and brain) according to TNM classification for breast cancer (Giuliano et al., 2018). The molecular type of participants is as follows: 47.2% of HR + HER2−, 31.8% of HER2+, and 21.0% of Triple negative. Demographic characteristics and health status of the participants are presented in Table 1.

**Table 1:** Demographic characteristics and health status of the participants.

	Number of patients	Percentage of patients
**Marital status**		
Married	177	90.8
Single, widowed, or separated	18	9.2
**Education**		
Middle school or below	109	55.9
High school or above	86	44.1
**Occupation**		
Unemployed or self-employed	44	22.6
Employed for wage	80	41.0
Retired	71	36.4
**Age**		
≤45	55	28.2
46–55	95	48.7
56–65	32	16.4
>65	13	6.7
**Perceived health status**		
Excellent	31	15.9
Great	105	53.8
Fair	51	26.2
Poor	8	4.1
**Length of treatment**		
Less than a year	32	16.4
1–3 years	71	36.4
3–5 years	38	19.5
5 years or more	54	27.7
**Length of delay**		
Less than a week	82	42.1
1–2 weeks	27	13.8
2–3 weeks	13	6.7
More than 3 weeks	73	37.5
**Breast cancer stage**		
Stage I	126	64.6
Stage II and III	60	30.7
Stage IV	9	4.7
**Molecular type**		
HR + HER2-	92	47.2
HER2+	62	31.8
Triple negative	41	21.0
**Type of treatment delayed**		
Surgery	3	1.5
Target Therapy	19	9.7
Endocrine Therapy	67	34.4
Chemotherapy	5	2.6
Radiotherapy	3	1.5
Post-operative check-ups	98	50.3

### The psychological effects of treatment delay

Independent samples one-way ANOVA was conducted to examine the effect of length of delay in treatment on general psychopathology score. The analysis revealed a significant effect of length of treatment delay on general psychopathology score, *F*(3, 191) = 56.46, *p* < 0.001, $\eta^{2}$ = 0.89. Follow-up multiple comparison analysis showed that participants who had their treatment delayed for more than 3 weeks (*M* = 1.77, *SD* = 0.46) reported a significantly higher general psychopathology score than participants whose delay in treatment was less than 1 week (*M* = 1.13, *SD* = 0.18, *p* < 0.001), 1 to 2 weeks (*M* = 1.27, *SD* = 0.18, *p* < 0.001), and 2 to 3 weeks (*M* = 1.37, *SD* = 0.15, *p* < 0.001), respectively. Then, we examined the effect of treatment delay on each factor of SCL-90 and found a statistically significant effect of length of treatment delay on somatization (*p* < 0.001), obsessive–compulsive disorder (*p* < 0.001), interpersonal sensitivity (*p* < 0.001), depression (*p* < 0.001) hostility (*p* < 0.001), phobic anxiety (*p* < 0.001), paranoid ideation (*p* < 0.01), and psychoticism (*p* < 0.001).

### The effect of treatment status on general psychological symptom

Independent samples t-test was performed to analyze how current status of the treatment affects general psychopathology score. Participants whose treatments continues to be delayed (*n* = 114), on average, reported an elevated general psychopathology score (*M* = 1.48, *SD* = 0.47) compared to participants whose treatments are resumed (*n* = 81, *M* = 1.30, *SD* = 0.34), and the difference was statistically significant, *t*(193) = 2.96, *p* = 0.003, *d* = 0.44, 95%Cl [0.06, 0.30].

### Main effect and interaction effect of breast cancer stage and length of treatment delayed on general psychopathology score

**Figure 1:**
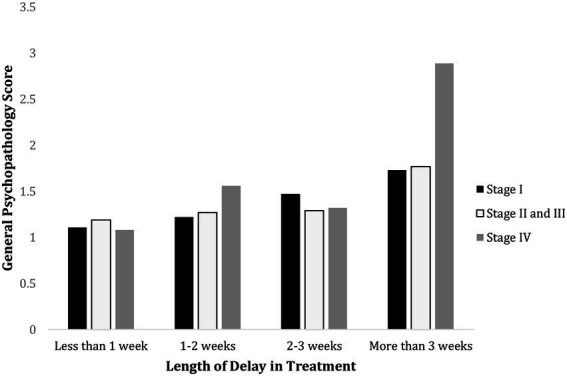
Psychological effect of treatment delay on breast cancer patients of different stages. This demonstrates that delay in treatment for more than 3 weeks elevates general psychopathology score. Patients with Stage IV breast cancer demonstrated significantly higher general psychopathology score when their treatment was delayed for more than 3 weeks than less than 1 week (*p* < 0.001), 1–2 weeks (*p* < 0.001), and 2–3 weeks (*p* < 0.001). Patients with Stage II and III breast cancer also showed significantly higher general psychopathology score with more than 3 weeks’ treatment delay than less than 1 week (*p* < 0.001), 1–2 weeks (*p* < 0.001), 2–3 weeks (*p* = 0.003). For patients at Stage I breast cancer, the general psychopathology score for a delay of more than 3 weeks was significantly higher than less than 1 week (*p* < 0.001) and 1–2 weeks (*p* = 0.035). However, no significant difference was found when treatment was delayed for more than 3 weeks and 2–3 weeks (*p* = 1.00).

3 (Cancer Stage: Stage I, Stage II & III, Stage IV) × 4 (Treatment Delay: less than 1 week, 1–2 weeks, 2–3 weeks, more than 3 weeks) between subjects ANOVA was performed to analyze the main effects of breast cancer stage and delay in treatment, and their interaction effect on SCL-90-R general psychopathology score. There was a significant main effect of breast cancer stage, such that participants with stage IV breast cancer reported higher general psychopathology score (*M* = 1.62 *SD* = 0.80) than participants with Stage I (*M* = 1.37, *SD* = 0.41) and Stage II and III breast cancer (*M* = 1.44, *SD* = 0.39), *F*(2, 183) = 4.19, *p* = 0.02, and $\;\eta_{p}{}^{2}\;$ = 0.05. There was also a significant main effect of delay in treatment, *F*(3, 183) = 39.39, *p* < 0.001, and $\;\eta_{p}{}^{2}\;$ = 0.65 on the general psychopathology score. These main effects were qualified for a statistically significant interaction effect, *F*(6, 183) = 4.07, *p* = 0.001, and $\;\eta_{p}{}^{2}\;$ = 0.13. To probe into this significant interaction effect, simple effect tests were performed to examine the effect of delay in treatment among Stage I, II and III, and IV breast cancer patients. The results are demonstrated in Figure 1.

### Main effect and interaction effect of delay in treatment and molecular types on general psychopathology scores

**Figure 2:**
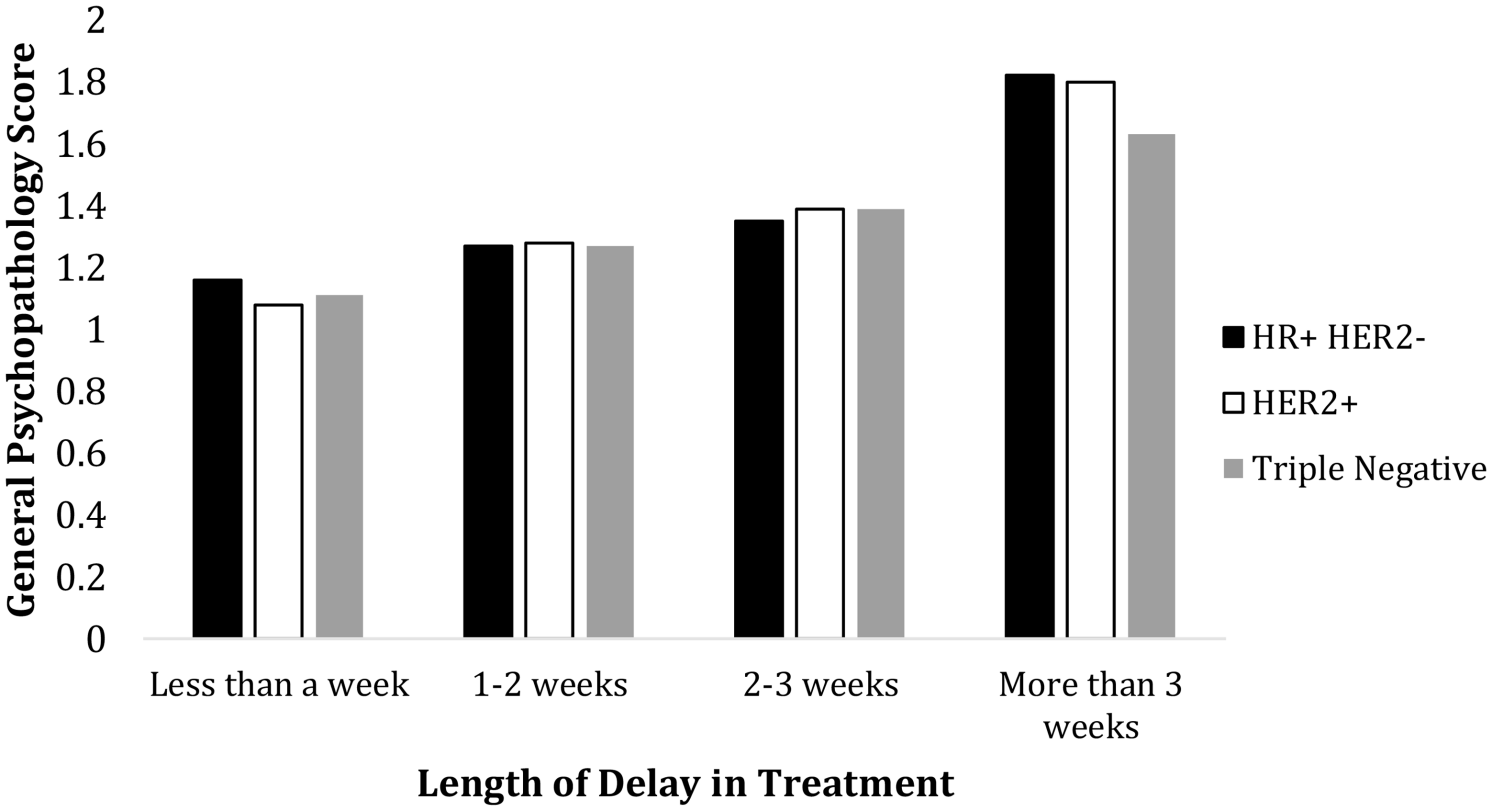
Psychological effect of treatment delay and molecular types. Molecular type did not have a significant effect on general psychopathology score, and there was no interaction of molecular type and length of treatment delay on the general psychological status.

3 (Molecular Type: HR+ HER2−, HER2+, Triple Negative) × 4 (Treatment Delay: less than 1 week, 1–2 weeks, 2–3 weeks, more than 3 weeks) between-subjects ANOVA was performed to test the main effects of molecular type and treatment delay and their interaction effect. Treatment delay had a significant main effect on the general psychopathology score, *F*(3, 183) = 45.76, *p* < 0.001, $\;\eta_{p}{}^{2}$ = 0.43. However, molecular type did not have a significant main effect on general psychopathology score *F*(2, 183) = 0.21, *p* = 0.81, $\;\eta_{p}{}^{2}\;$ = 0.002. Nor was there an interaction effect of treatment delay and molecular types on general psychology status, *F*(6,183) = 0.43, *p* = 0.86, $\;\eta_{p}{}^{2}$ = 0.01. The results are shown in Figure 2.

### General psychopathology scores for each type of treatment delayed

**Figure 3:**
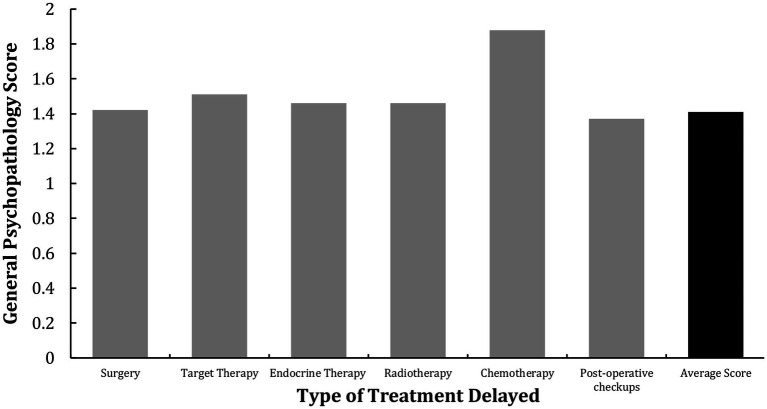
The effect of delayed treatment type on general psychopathology score. The delay of chemotherapy had a significant effect on breast cancer patients’ general psychopathology score.

Independent samples t-tests were performed to examine the effect of the type of treatment delayed on the general psychopathology score. The results showed that except for chemotherapy, the delay of which significantly elevated general psychopathology score, *t*(193) = −2.55, *p* = 0.01, *d* = −0.37, 95%CI = [−5.44, 0.44], treatment delay of other types did not have a significant effect on general psychological status (*p* > 0.05). The results are demonstrated in Figure 3.

### The predictive effect of age, perceived health status, current treatment status, and length of treatment delay on general psychological status

The results of multiple linear regression analyses are shown in Table 2. SCL-dimension scores were regressed on age, education, perceived health status, molecular type, breast cancer stage, COVID effect on one’s city, current treatment status, and length of delay. Perceived health status (*p* = 0.006), current treatment status (*p* = 0.043) and the length of treatment delay (*p* < 0.001) are the only variables that were statistically associated with general psychopathology score. Poorer perceived health status, in addition to continued and longer treatment delay, were associated with higher general psychopathology score. Furthermore, longer treatment delay was associated with higher somatization (*p* < 0.001), obsessive–compulsive (*p* < 0.001), interpersonal sensitivity (*p* < 0.001), depression (*p* < 0.001), hostility (*p* < 0.001), phobic anxiety (*p* < 0.001), paranoid ideation (*p* < 0.001), and psychoticism (*p* < 0.001). Additionally, younger age was associated with higher interpersonal sensitivity (*p* = 0.025), depression (*p* = 0.046), and hostility (*p* = 0.01).

**Table 2:** Results of multiple regression analyses.

SCL-90 dimension		Age	Education	Perceived health status	Cancer stage	Molecular type	COVID effect on city	Current treatment status	Length of delay
General index	β	−0.04	−0.004	0.10**	0.03	−0.03	−0.02	−0.06*	0.19***
	SE	0.03	0.05	0.03	0.04	0.02	0.05	0.03	0.02
Somatization	β	0.35	−0.72	1.82***	0.50	−0.74	0.12	−1.36**	2.36***
	SE	0.45	0.74	0.55	0.66	0.34	0.73	0.43	0.28
Obsessive–compulsive	β	−0.50	0.08	0.92	0.02	−0.28	−1.01	−0.48	2.67***
	SE	0.44	0.73	0.54	0.65	0.33	0.72	2.67	0.28
Interpersonal sensitivity	β	−0.82*	0.22	0.93*	0.52	−0.29	−0.02	−0.47	1.90***
	SE	0.36	0.60	0.44	0.54	0.27	0.59	0.35	0.23
Depression	β	−1.00*	−0.34	1.91**	0.57	−0.04	0.39	−0.80	3.37***
	SE	0.50	0.82	0.61	0.74	0.37	0.81	0.48	0.31
Anxiety	β	−0.04	−0.12	0.18	0.08	0.07	−0.10	−0.22	0.02
	SE	0.20	0.34	0.25	0.30	0.15	0.33	0.20	0.13
Hostility	β	−0.68**	−0.12	0.41	0.20	−0.41	−0.32	−0.23	1.10***
	SE	0.26	0.43	0.32	0.38	0.19	0.42	0.25	0.16
Phobic anxiety	β	−0.10	−0.04	0.40	0.11	−0.13	−0.34	−0.21	1.28***
	SE	0.28	0.47	0.35	0.42	0.21	0.46	0.27	0.18
Paranoid ideation	β	−0.11	0.14	0.17	0.13	−0.13	−0.41	−0.23	0.99***
	SE	0.21	0.35	0.26	0.31	0.16	0.34	0.20	0.13
Psychoticism	β	−0.44	0.21	0.57	0.27	−0.09	−0.42	−0.57*	1.71***
	SE	0.30	0.49	0.36	0.44	0.22	0.48	0.29	0.19

Reference categories: aged 45 years old or below; education level is middle school or below; perceived health status is excellent; Cancer is at Stage 1; Molecular type is HR + HER2-; COVID had no effect on city; current treatment status is delayed, and length of treatment delay is less than a week.

* *p* ≤ 0.05;

** *p* ≤ 0.01;

*** *p* ≤ 0.001.

## Discussion

COVID has spread to more than 70 countries and has become a global health crisis. The pandemic dramatically impacted people worldwide and has posed an unprecedented challenge to breast cancer patients’ psychosocial well-being. Previous studies have applied the SCL-90-R psychometric measures to examine the psychological impact of COVID on the general public and cancer survivors. However, this study extended beyond previous literature and investigated the separate and joint effects of breast cancer stage, length and type of treatment delay, and molecular type on patients’ psychological status.

A major finding in this study is that cancer survivors at an advanced stage of breast cancer are more likely to experience psychological symptoms with longer treatment delay, and cancer patients whose treatments continues to be delayed reported elevated psychological symptoms than individuals whose treatment are resumed, regardless of treatment type. Additionally, a treatment delay of more than 3 weeks may exacerbate breast cancer patients’ psychological symptoms, whereas a short-term delay of less than 3 weeks is less likely to have a significant effect on one’s mental well-being. Therefore, to protect the physical and mental well-being of advanced-stage breast cancer patients during trying times such as COVID, delay in cancer treatment should not exceed 3 weeks. Also, our results suggest that perceived health status more significantly predicts one’s psychological well-being than cancer stage. This finding has an essential implication as it highlights the importance of building health confidence in breast cancer care.

Given that strengthening health confidence may improve one’s perceived health status, future research should develop interventions to improve cancer survivors’ health confidence. This study also found that younger age was associated with higher interpersonal sensitivity and hostility. These results are in line with empirical research findings that altered body image due to cancer treatment leads to stronger body image concerns for younger women (Manier et al., 2018), which tends to reduce their self-esteem and, in turn, leads to fear of interpersonal interaction. This finding might be valuable for caregivers, who should provide breast cancer survivors with positive affirmations to help them regain confidence and maintain meaningful social relationships with trustworthy friends.

The current study found a significant interaction effect of breast cancer stage and length of treatment delay on the general psychological health, such that longer treatment delay elevates participants’ psychological symptoms, especially for those at more advanced stages. However, our data are preliminary, as the study’s participants only consist of a subpopulation of breast cancer patients in China, whose post-COVID treatment experience may not be representative of the experience of breast cancer patients in different regions of China. What is more, the sample size for some of our subgroups (i.e., Stage IV breast cancer) are relatively small, which might have weakened the power of some of our analyses. A follow-up study could sample a larger number of breast cancer patients from various regions and religions to examine whether factors such as region-based differences and beliefs affect people’s psychological health.

Additionally, research has shown that the median age of breast cancer onset for Asian women is 56 (Stapleton et al., 2018). However, a disproportionately large percentage of this study’s participants were 55 years or below. This might be due to the fact that the survey was administered online, and older people, in general, have worse eyesight and are less technologically adept than their younger counterparts. Thereby, although some people would be willing to participate in the study if it was administered in the paper format or through a phone call interview, they did not attempt the online survey because of technological challenges. Some other people might have attempted the survey but could not finish due to fatigue, difficulty concentrating, or poor eyesight. Future replication studies could address the limitations by administering the survey in different formats based on participants’ age and preferences. For people who have difficulties completing the survey online, a phone call survey could be offered as an alternative.

Furthermore, some participants might face stressors in their lives that others do not. For example, in addition to their cancer diagnosis and COVID-related existential threat, some people may be impacted by additional stressors such as financial insecurity, loss of a loved one, or lack of support from their family members. Thus, some breast cancer patients’ elevated general psychopathology scores might not be the sole effect of their advanced stage or COVID-related treatment delay. A follow-up study could compare participants’ psychological symptoms at the peak of the pandemic with the baseline level when COVID ends and the stressors due to cancer delays are removed to examine the extent to which delay in treatment contributes to participants’ aggravated psychological symptoms.

## Conclusion

In summary, our study found that longer treatment delay is associated with higher somatization obsessive–compulsive, interpersonal sensitivity, depression, hostility, phobic anxiety, paranoid ideation, and psychoticism among the participants, and treatment delay of more than 3 weeks leads to more acute psychological symptoms, especially for breast cancer patients at more advance stages. However, the results of the study is preliminary and left several questions unanswered. To rule out alternative explanations for some of our results, follow-up research should recruit participants from more diverse backgrounds and compare participants’ psychological status with baseline level when stressors due to COVID-induced treatment delays are eliminated.

## Data availability statement

The original contributions presented in the study are included in the article/supplementary material, further inquiries can be directed to the corresponding author.

## Ethics statement

The studies involving human participants were reviewed and approved by Xijing Hospital. The patients/participants provided their written informed consent to participate in this study.

## Author contributions

NL conceived and designed the analysis. JY and HW collected the data. YY and CY contributed data and analysis tools. WM and YW performed the analysis and wrote the paper. All authors contributed to the article and approved the submitted version.

## Conflict of interest

The authors declare that the research was conducted in the absence of any commercial or financial relationships that could be construed as a potential conflict of interest.

## Publisher’s note

All claims expressed in this article are solely those of the authors and do not necessarily represent those of their affiliated organizations, or those of the publisher, the editors and the reviewers. Any product that may be evaluated in this article, or claim that may be made by its manufacturer, is not guaranteed or endorsed by the publisher.
